# You are how (and where) you search? Comparative analysis of web search behavior using web tracking data

**DOI:** 10.1007/s42001-023-00208-9

**Published:** 2023-05-03

**Authors:** Aleksandra Urman, Mykola Makhortykh

**Affiliations:** 1 University of Zurich, Zurich, Switzerland; 2 Department of Communication and Media Studies, University of Bern, Bern, Switzerland

**Keywords:** Web search behavior, Web tracking, Survey, Germany, Switzerland, Search engines

## Abstract

In this article, we conduct a comparative analysis of web search behaviors in Switzerland and Germany. For this aim, we rely on a combination of web tracking data and survey data collected over a period of 2 months from users in Germany (*n* = 558) and Switzerland (*n* = 563). We find that web search accounts for 13% of all desktop browsing, with the share being higher in Switzerland than in Germany. In over 50% of cases users clicked on the first search result, with over 97% of all clicks being made on the first page of search outputs. Most users rely on Google when conducting searches, with some differences observed in users’ preferences for other engines across demographic groups. Further, we observe differences in the temporal patterns of web search use between women and men, marking the necessity of disaggregating data by gender in observational studies regarding online information seeking behaviors. Our findings highlight the contextual differences in web search behavior across countries and demographic groups that should be taken into account when examining search behavior and the potential effects of web search result quality on societies and individuals.

## Introduction

Web search engines are ubiquitous nowadays and act as major information gate-keepers in high-choice media environments. Google alone handled around 6.9 billion queries per day in 2020 [23] with an average user of Google.com turning to the site 18.15 times per day as of April 2021 [2]. The numbers are staggering, especially given that Google is just one of the search engines—though the dominant one on most markets. Furthermore, search engines are highly trusted by their users: according to Edelman Trust Barometer [3], in 2020 search engines were reported to be the most trusted information source globally.

Given the importance of search engines for shaping public opinion, it is crucial to understand users’ web search behaviors. Yet, our knowledge in this context remains limited and primarily relies on two types of data: eye tracking [19, 24] and search engine transaction log data [10, 28]. Both of these data sources have their limitations: eye-tracking studies typically rely on small user samples and can hardly be generalized to broader populations. Log-based studies capture the behavior of the large groups of users, but on the aggregate level, thus limiting the possibilities for inferring the impact of individual characteristics on how users search for information. In addition, log-based studies cannot reliably infer the connection between search results ranking and user behavior, because researchers cannot, in retrospect, identify how search results were ranked and presented to individual users due to effects of search personalization [9] and randomization [17].

In the present study, we address these limitations and explore *the contextual differences in the patterns of web search behavior* using the combination of web tracking [4] and survey-based demographic data collected in Germany and Switzerland in spring 2020. Web tracking data includes information on user desktop-based browsing behavior along with the actual HTMLs of the browsed content. By acquiring HTMLs of pages viewed by the users, we can infer the exact composition and ranking of web search results users were exposed to and, consequently, find out which of these results they clicked on. Such a combination of data allows us to address several gaps in the existing scholarship on web search behavior. First, we scrutinize the effect of individual demographic characteristics on search behavior using a large sample of users. Second, unlike earlier log-based search behavior studies, which were focused on single-country populations (usually, the US), our study offers a comparative perspective and goes beyond the US context. Third, we examine the user clicking behavior in relation to web search results ranking on the topic that typically rely on smaller samples and are carried out in lab settings.

Specifically, we address the following research questions: (1) How frequently do users with different demographic characteristics and socioeconomic status use search engines? (2) What are the temporal patterns of web search use and do they differ by demographics? (3) Are there demographic or socioeconomic status-based differences in the choice of specific search engines (i.e., Google/Bing/other)? (4) How does the rank of a search result relate to the clicking behavior of users with different demographics? We also examine country-level differences in relation to each of the four questions and discuss their practical implications.

## Related work

Studies on web search behavior to date have relied on either of the two data source types: eye tracking and search engine transaction log data.

*Eye-tracking-based studies* are typically conducted on smaller not demographically representative samples, and within lab settings. The advantage of such studies is that they allow examining user attention patterns in the context of web search and, for instance, exploring the relation between the ranking of search results and users’ clicking behaviors. In one of the earliest studies [7], the authors have examined users’ attention and clicking patterns based on the student sample (*n* = 36), and found that top-ranked search results receive disproportionately more attention and clicks than low-ranked ones. This finding was corroborated in numerous further studies [7, 12, 19, 24].

Eye-tracking studies have also investigated the impact of additional factors on search result selection. For instance, two studies that used a small (*n* = 18) sample of users of diverse ages and occupations [13] and a student sample (*n* = 22) [20] from the US found that clicking decisions are influenced not only by ranking but also perceived relevance of search results. A replication of the latter study [25] conducted circa 10 years later on a student sample (*n* = 28) in Germany has found similar effects thus indicating the stability of observed effects across time and different national contexts.

Despite providing important insights into user search behavior, eye-tracking studies have a number of limitations, in particular their limited scalability. While some solutions for scaling are being offered in recent years—e.g., eye tracking via webcam devices [21]—their precision remains lower than that of more conventional lab-based eye trackers [10]. Owing to the scalability problem, eye-tracking studies are based on the small samples which are not demographically representative and, often, are made of students recruited in the US. This leads to a limited generalizability of eye-tracking-based findings: it is unclear whether users with different demographics search the web in similar ways, and whether there are country-level differences in how they do it.

*Transaction log-based studies* allow examining web search behavior at scale. One of the earliest studies utilizing transaction log data [26] found that users tend to type in short queries and rarely navigate beyond page 1 of the search results. Similar findings were reported by authors of a 1-week-long study based on a Korean search engine Naver [22].

Log data has also been utilized to examine temporal aspects of web search [31] and the patterns of search query usage [30]. Such studies allow inferring real-life web search usage patterns and are based on the large data samples. However, log-based studies also have several limitations. First, due to the difficulty of obtaining search logs data owned by proprietary companies, most of the studies focus on single search engines. It undermines the generalizability of their findings since usage patterns can be affected by the differences in search engine interfaces or the differences in the demographics of their users. Even studies, such as [11] that analyze log data from multiple search engines cannot match the users across these engines, which prevents them from examining if the same users utilize multiple different engines and, if so, whether and how their behavior is different depending on the engine.

The absence of reliable demographic data about the users is another limitation of log-based studies. Such data are sometimes available on users’ gender and age, but not on other variables such as education or income level that can only be inferred by the researchers [30]. However, such inference-based studies are rare. Third, log-based data are inherently noisy because search requests might be executed not only by human users but also by bots, and it is difficult to differentiate between organic and automated requests [12]. Finally, the transaction log data typically does not allow tracing the position of the search results a user clicked on and the only ranking-related parameter available is the search result page on which a user selected a result.

The aforementioned limitations of both approaches can be addressed by utilizing web tracking data that includes full HTMLs of the pages browsed by users and is combined with survey data. Unlike eye tracking and log-based data, web tracking is scalable and allows observing user behavior in real-life circumstances. Furthermore, it allows to reliably know users’ demographics (from the survey), observe user behavior across multiple search engines, make sure that the data comes from real users and not bots and infer the exact position of the result a user clicked on. Thus, this approach allows combining the strengths of both approaches previously utilized to measure web search behavior, while overcoming their limitations.

## Data and methods

### Data

To collect the data, we recruited a sample of Internet-using participants in the age range of 18–75 years from Germany and German-speaking Switzerland. The recruitment was conducted via the market research company Demoscope in early March 2020 using online access panels with 200,000 members (Germany) and 35,000 members (Switzerland). Participants were randomly selected according to gender, age, and education quotas to construct demographically representative samples of the German and the German-speaking Swiss population. For Germany, the region of residence (West vs. East) was used as an additional sampling criterion.

The selected participants were invited to fill out the survey, which was completed by 1952 participants in Germany and 1297 in Switzerland. As a requirement to take part in the survey, participants were asked whether they agreed to participate in the online tracking study using a browser extension that records their online behavior. While agreement to be tracked was required to partake in the survey, participants were informed that they could opt out from being tracked at any time.

After agreeing to participate in online tracking, each participant received a link to extensions for desktop versions of Chrome and Firefox browsers. The extensions were designed specifically for the project and captured full HTMLs of web content appearing in the browser, where the extension was installed [4]. The captured HTML content together with the URL address of the page from which it was captured were sent to the remote server, where data were encrypted and stored.

To protect participant privacy, the extensions were supplemented by a "hard" denylist (i.e. a list of websites whose content was not captured and visits to which were not recorded; this included insurance companies, medical services, pornography websites, bank websites, messengers, and e-mail services) and a "soft" denylist (i.e. a list of websites whose content was not captured, but the visits to which were recorded; the list included commercial websites). Participants were also provided with the possibility to switch browser extensions to ‘private mode’, where no content was captured, so they could browse privately if they felt the need to. This was done to ensure participants’ privacy and alleviate their potential concerns regarding participation in the tracking study [18].

Out of the original sample of participants expressing agreement to being tracked, 587 (Germany) and 601 (Switzerland) participants had successfully registered at least one website visit by the end of the tracking period (March 17 to May 26 2020). Our sample consists only of those who registered at least one web search—563 participants in Switzerland and 558 in Germany. The participants were asked about their age, gender, education and income. The reported levels of education were collapsed into three subgroups to ensure comparability between the two countries: obligatory school only, full secondary education, tertiary education. Participants also reported their monthly income (according to predefined income breaks, different for Switzerland and Germany due to the differences in the overall income levels between the two countries).

The demographic distributions in the samples are as follows: self-reported gender: CH—43.8% female, DE—44.8% female; age: CH—mean = 43.8, median = 42, DE—mean = 49.3, median = 51; education: CH—3.6% obligatory education, 54.9%—full secondary education, 41.6%—tertiary education, for DE corresponding numbers are 12.9, 51.3, 35.8%; income: CH—7.2% not reported, 37.7% below 3999CHF, 37.1% between 4000 and 6999 CHF, 17.9% above 7000 CHF; DE—3.1% not reported, 48.4% below 1999 EUR, 44.8% from 2000 to 4999 EUR, 3.8% above 5000 EUR. The resulting samples deviate from the actual composition of the population of the corresponding countries—e.g., in our samples, men and people with higher levels of education are over-represented as compared to the actual population; such skewness in the participants willing to participate in web tracking studies was observed in our case and with another study conducted in Germany using a similar recruitment procedure in 2021 [6]. As extensively discussed in [6], this is linked to different concerns different groups of participants had about the tracker. For this study, it is important to note that the main reasons of the participants for dropping out of the study and not participating in tracking were concerns about their perceived computer skills and the safety of their devices and data [6]; hence, our findings might under-represent the behaviors of more privacy−conscious participants and those who perceive themselves as less technologically savvy. For the potential ways to mitigate these sampling issues in future studies, interested readers can refer to Ref. [6].

### Methods

To filter out web search visits from the overall tracking data, we compiled a list of search engines commonly utilized by European users as indicated by sources such as (Barometer 2021). The list included the following engines: Google, AOL, Bing, DuckDuckGo (DDG), Ecosia, Gigablast, Metager, Qwant, Swisscows, Yahoo, Yandex. Then, we extracted all visits to respective domains and filtered the data by subdomains using URL parts that would point to a domain service other than web search (e.g., Google Photos in the case of Google). Then, we calculated the share of visits to image search, video search as well as news search among all search traffic. In total, we have recorded 348,018 user visits to text, image and video search across all engines combined. The results have demonstrated that visits to these services are infrequent: image search accounted for 0.2% of search engine traffic, video search for 0.06%. Thus, we focused on text search only as it accounted for over 99.7% of all search traffic.

After filtering out text search results, we merged the users’ tracking data with their demographic data obtained via the survey. This merged data was used in the next steps of the analysis. Each step was performed separately for the German and the Swiss subsamples, with comparisons drawn between the two.

RQ1: How frequently do users with different demographic characteristics and socio-economic status use search engines?

To establish how frequently users with different demographic characteristics utilize web search, we computed descriptive statistics about the average proportion of visits to web search engines to the overall number of visits tracked and average number of search queries executed daily by users from different age and gendergroups.

RQ2: What are the temporal patterns of web search use and do they differ by demographics?

To examine temporal patterns of web search and their differences by country and demographics, we have calculated the frequency of web search use by the day of the week and time of the day (morning = 6am to 12 pm; afternoon = 12 pm to 6 pm; evening = 6 pm to midnight; night = midnight to 6am). We then compared the patterns that emerged.

RQ3: Are there demographic or socio-economic status-based differences in the choice of specific search engines (i.e., Google/Bing/other)?

To assess the differences in the user preferences for specific search engines by demographics, we calculated descriptive statistics across engines. First, we calculated the share of each search engine in overall web search traffic in our sample. Then, for the search engines that accounted for at least 1% of search traffic in either of the two (German and Swiss) samples, we calculated the share of users in each demographic (gender; age) group that used the engine at least once during the observation period. Then, among those users who used an engine at least once, we calculated the average share of search traffic each user (disaggregated by gender and age groups) devoted to a specific engine to assess the strength of users’ preferences towards specific engines.

RQ4: How does the rank of a search result relate to the clicking behavior of users with different demographics?

To establish the association between result ranking and clicking behavior, we have extracted links for organic text results from the HTML of pages browsed by the users for Google and Bing. We focused on these two engines due to them being used most frequently by users (see “Results”, subsection 1). We extracted the links in the same order as they appeared in users’ browsers. Then, we matched this data to the URLs accessed by the users after each search engine visit to infer which search result users clicked on. Based on this, we calculated summary statistics about the share of clicks different search pages and differently ranked results received.

### Limitations

Before we proceed to the description of the results, it is important to note the two major limitations of our study as they have implications for the interpretation of our findings. First, we examine only desktop-based browsing behavior because mobile-based tracking is notoriously complex to implement, especially in a way that would make mobile data comparable with the desktop one [4]. This is an important limitation given that mobile devices account for roughly half of the global internet traffic. Also, given that users’ desktop- and mobile-based behaviors might differ, our findings have to be interpreted only in the context of desktop browsing. Future work focusing on mobile browsing is necessary to investigate the differences in desktop- and mobile-based searching behaviors. Another limitation is that our data collection happened in the beginning of the COVID-19 pandemic and thus coincided with the lockdowns in both, Germany and Switzerland. In light of this, our findings, especially those related to the temporal patterns of web search, need to be interpreted with caution, because it is difficult to evaluate whether and how much users’ search behaviors during the lockdowns might have been different from those in routine times.

## Results

RQ1: Frequency of search engine use by demographics

On average, participants used text search 8.8 times per day (see Table 1). There were no major differences in the number of average daily searches between the two countries: in Switzerland, users engaged in web search on average 8.76 times per day, while in Germany 8.84 times per day. However, there were differences in the ratio of web search visits to the overall number of visits tracked. In the overall sample, on average each user had 13% of their total tracked browsing devoted to web search. In the German sample this number was 10.4%, while in the Swiss one—15.5%. Thus though the users from both countries executed similar numbers of searches on a daily basis, in Switzerland web search accounted for a higher share of total internet browsing. This is in line with the discrepancy in the average number of pages browsed in total: 2322.1 pages in Switzerland versus 3833.7 in Germany.

**Table 1:** Average ratio of web search visits to the overall number of visits tracked and average number of searches executed daily per user, differentiated by country subsamples and demographic groups

Country sample	Variable	Overall	Women	Men	Age 18–34	Age 35–54	Age 55+
Germany	Share of search in browsing	10.4%, 95% CI [9.66%, 11.14%]	10.6%, 95% CI [9.84%, 11.4%]	10.2%, 95% CI [9.52%, 10.9%]	12%, 95% CI [11%, 12.7%]	10.2%, 95% CI [9.49%, 10.8%]	10%, 95% CI [9.3%, 10.8%]
	Average number of searches daily	8.84, 95% CI [8.03, 9.64]	7.71, 95% CI [6.98, 8.44]	9.75, 95% CI [8.89, 9.6]	9.52, 95% CI [8.5, 10.53]	9.72, 95% CI [8.62, 10.22]	7.96, 95% CI [7.26, 8.66]
Switzerland	Share of search in browsing	15.5%, 95% CI [14.5%, 16.5%]	17.1%, 95% CI [16.1%, 18.1%]	14.2%, 95%CI [13.3%, 15.2%	18.6%, 95% CI [17.5%, 19.7%]	14.6%, 95% CI [13.6%, 15.5%]	12.8%, 95% CI [12%, 13.6%]
	Average number of searches daily	8.76, 95% CI [8.04, 9.48]	8.51, 95% CI [7.81, 9.21]	8.95, 95% CI [8.21, 9.69]	11.8, 95% CI [10.84, 12.77]	7.65, 95% CI [7.09, 8.21]	6.33, 95% CI [5.96, 6.69]

RQ2: Temporal patterns of web search

**Fig. 1:**
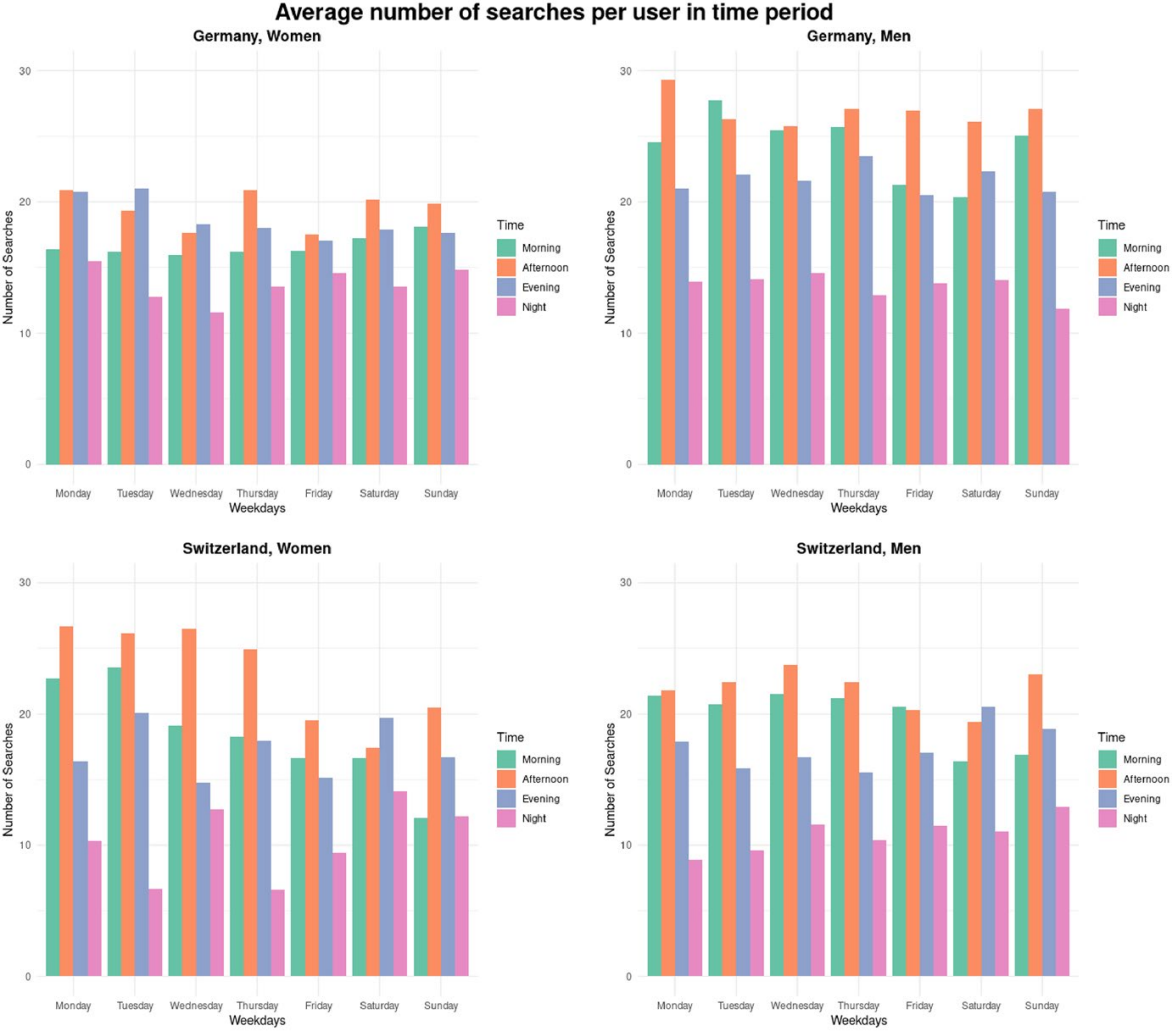
Average number of searches executed per user in weekday and time of day periods, disaggregated by country and gender

The analysis of temporal patterns of web search reveals major differences with regard to when users of different gender use search engines. The average numbers of searches executed per user on each weekday and time of the day periods are presented in Fig. 1.

In both, Switzerland and Germany, men’s search patterns are stable throughout the week, with the most active search times being morning and afternoon. In the evenings, searching is less prevalent than in the mornings and afternoons, followed by a major drop in searching at night. Women’s search patterns, however, are different. In Germany, they are similarly to men’s consistent throughout the week but women tend to search way less than men in the mornings and afternoons, slightly less in the evenings, and with the same intensity in the night.

The differences in German women’s search activity by the time of the day are way less drastic than in men’s search activity. In Switzerland, on the other hand, time-of-day-based differences in women’s search activity are similar to those observed for men. However, Swiss women’s searches in the mornings and afternoons are unevenly distributed throughout the week: from Monday to Thursday women actively use web search in the afternoons, but from Friday to Sunday their search activity in the afternoon is reduced as compared to the first part of the week. Morning search activity among Swiss women decreases progressively from the start to the end of the week.

The observed differences among women and men in both countries are the most pronounced among younger (18–45 years old) part of the populations, suggesting that they might have to do with the life circumstances of younger women such as, potentially, child-bearing and child-caring duties. However, based on the available data, it is hardly possible to verify this interpretation. Regardless of the reasons behind the observed discrepancies, our findings once again highlight the necessity of disaggregating data about information behavior by gender [5] to grasp the behavioral patterns of all parts of the population properly.

RQ3: Usage of specific search engines across demographic groups

Google largely dominates search traffic in both country subsamples, being slightly more prevalent in Switzerland than in Germany, as demonstrated in Table 2. In Germany, it is followed by Bing with the latter accounting for 7.4% of all traffic—substantially lower than Google but more than 3 times higher than all other engines in either of the country subsamples.

**Table 2:** Share of searches within a specific search engine among all searches from all users by the country subsample

Country	Google	Bing	Ecosia	DDG	Other (total)
Germany	88%	7.4%	2.5%	0.5%	1.6%
Switzerland	94.4%	1.5%	2%	1.3%	0.8%

The numbers are reported only for specific search engines for which the share is above 1% in one of the subsamples. The share of all other search engines is aggregated as "Other"

In Switzerland, Bing is less prominent, with Ecosia being the second most popular engine that accounts for 2% of search traffic. The only other engine that received at least 1% of the traffic in either of the samples is DuckDuckGo (DDG) with 1.3% of traffic in Switzerland. These findings are in line with the reports made by companies monitoring global search traffic on country-level such as Statcounter [27].

In Table 3, we report the observations on the share of participants who used each search engine at least once disaggregated by demographics. It comes as no surprise that Google was used at least once by almost all participants. The second most popular engine by the share of users who turned to it at least once is Bing. It was used at least once by 17.7% of German participants and 10.7% of Swiss participants, with the share of men turning to Bing being higher than the share of women in both cases. The patterns of Bing usage by age groups, however, vary between the two countries with it being more prevalent among elder users in Germany and among younger users in Switzerland. The other engines were used at least once by a marginal share of users—less than 5% across both subsamples and all demographic groups. We observe no consistent gender/age patterns with regard to Ecosia and DuckDuckGo usage.

**Table 3:** Share of participants who used a specific search engine at least once by country, gender and age group

Country sample	Engine	Overall	Women	Men	Age 18–34	Age 35–54	Age 55+
Germany	Google	96.6%	96.4%	96.8%	96.8%	98.3%	94.8%
	Bing	17.7%	15.6%	19.5%	11.8%	18.8%	19.1%
	Ecosia	4.5%	4%	4.9%	4.3%	4.3%	4.9%
	DDG	1.8%	2%	1.6%	0%	2.1%	2.8%
Switzerland	Google	97.9%	98%	97.8%	97.6%	98.9%	97.1%
	Bing	10.7%	8.9%	12%	11.2%	10.4%	10.3%
	Ecosia	3.2%	4.5%	2.2%	4.9%	1.1%	3.4%
	DDG	3.4%	2.4%	4.1%	3.4%	2.8%	4%

In Table 5, we report average shares of search traffic via a given search engine per user among participants who used the engine at least once, and in Table 4, the share of users who used more than one search engine. This way we can assess how strong are participants’ preferences for specific search engines—i.e., whether participants from different demographic groups tend to search within one search engine almost exclusively or engage in search across different engines, and whether the preference strength varies from one engine to another.

**Table 4:** Share of users in each demographic group who used more than one search engine

Country	Overall	Women	Men	Age 18–34	Age 35–54	Age 55+
Germany	26.9%	21.6%	31.2%	16%	29.9%	28.3%
Switzerland	23.6%	21.5%	25.2%	20.4%	22.5%	23.6%

In both samples, around a quarter of all participants used more than one search engine; the share of such participants tends to be higher among men and older users in both cases (Table 4). We observe that Google tends to be the default search engine for all demographic groups in both countries, with participants who used it at least once directing around 90% of their search traffic there. The participants’ preferences were less strong in their usage of other search engines. Though Bing has been used at least once by a larger proportion of participants than the other engines (Table 5), most of the demographic groups tend to direct only around 30–40% of their search traffic through this engine, thus indicating the absence of a strong preference for it. Those who used Ecosia or DuckDuckGo tend to have a stronger preference for these engines, directing a 50–60% of search through them, though the strength of the preference is clearly way lower than that of Google users. It has to be noted, however, that as overall relatively small shares of users used search engines other than Google at least once, Confidence Intervals corresponding to the % of search traffic they directed through these engines are rather wide, and there was a lot of variability on this among individual users.

RQ4: Ranking of search results and user clicking behavior

**Table 5:** Mean share of usage of specific search engines in all search visits per user, among participants who used a specific engine at least once

Country sample	Engine	Overall	Women	Men	Age 18–34	Age 35–54	Age 55+
Germany	Google	90.14%, 95% CI [92.25%, 88.02%]	91.35%%, 95% CI [89.35%, 93.35%]	89.15%, 95% CI [86.96%, 91.35%]	93.74%, 95% CI [92%, 95.5%]	90.48%, 95% CI [88.46%, 92.5%]	88.26%, 95% CI [85.94%, 90.59%]
	Bing	37.07%, 95% CI [29.15%, 45%]	42.48%, 95% CI [34.34%, 50.61%]	33.56%, 95% CI [25.79%, 41.33%]	48.15%, 95% CI [40.23%, 56.06%]	26.93%, 95% CI [20.26%, 33.6%]	44.45%, 95% CI [35.7%, 53.2%]
	Ecosia	61.07%, 95% CI [46.13%, 76%]	66.69%, 95% CI [52.49%, 80.88%]	57.32%, 95% CI [41.6%, 73.04%]	75.86%, 95% CI [61.1%, 90.62%]	54.59%, 95% CI [40.33%, 68.85%]	61.58%, 95% CI [45.23%, 77.98%]
	DDG	59.28%, 95% CI [34.99%, 83.56%]	61.87%, 95% CI [39.17%, 84.58%]	56.67%, 95% CI [28.3%, 85.04%]	—	62.49%, 95% CI [39.94%, 85.03%]	56.06%, 95% CI [27.63%, 84.49%]
Switzerland	Google	94.07%, 95% CI [92.41%, 95.73%]	94.5%, 95% CI [93.31%, 96.42%]	93.5%, 95% CI [91.72%, 95.19%]	94.32%, 95% CI [92.64%, 96.01%]	95.91%, 95% CI [94.58%, 97.24%]	91.83%, 95% CI [89.92%, 93.73%]
	Bing	28.61%, 95% CI [19.18%, 38.03%]	30.35%, 95% CI [21.28%, 39.41%]	27.6%, 95% CI [17.86%, 37.34]	22.75%, 95% CI [13.56%, 31.94%]	17%, 95% CI [9.33%, 24.68%]	48.34%, 95% CI [38.45%, 58.24%]
	Ecosia	55.72%, 95% CI [36.22%, 75.21%]	52.63%, 95% CI [32.58%, 72.68%]	60.57%, 95% CI [40.64%, 80.5%]	58.91%, 95% CI [40.66%, 77.16%]	58.02%, 95% CI [33.43%, 82.61%]	49.64%, 95% CI [26.03%, 73.25%]
	DDG	52%, 95% CI [32.2%, 71.81%]	60.13%, 95% CI [38.85%, 81.41%]	48.25%, 95% CI [28.51%, 68%]	62.12%, 95% CI [41.1%, 83.14%]	49.39%, 95% CI [32.8%, 65.7%]	43.76%, 95% CI [21.17%, 66.34%]

We have examined users’ clicking behavior on Google and Bing—the two engines which were visited most frequently. We found that on both engines participants clicked disproportionately more on top results. On Google, 97.11% of all clicks were associated with the results displayed on the first page; on Bing this percentage was even higher—99.49%.

Even within the first page, users’ clicks are distributed unequally. On Google, 51.3% of all clicks were associated with the very first result, followed by 15.68% of clicks on the second result, 9.23% on the third, 5.93% on the fourth, 4.21% on the fifth. Hence, top-5 search results accounted for over 86% of all clicks. On Bing, the corresponding distribution of clicks for the top-5 results is as follows: 52.18%; 19.72%; 9.24%; 7.35%; 3.57%. Thus, on Bing top-5 results received around 92% of all clicks. We did not observe major differences in the users’ clicking behavior between the two engines, suggesting that it does not depend on the engine, at least when both engines have a similar (i.e., results presented in a ranked list) interface.

We found that there seems to be a difference in the behavior of the users from two countries as in the Swiss subsample tendency to click on high-ranked results is even more pronounced than in the German one. The mean of the average ranking of the results clicked per user for Switzerland is 2.49 (median = 2.3), and in Germany the corresponding values are 3.13 and 2.55.

## Discussion

Our analysis shows substantial differences across countries in all four aspects of web search behavior and highlights the need for more comparative research. Since behaviors of users from Germany and Switzerland, two geographically and, in part, culturally proximate countries, are vastly different, it is reasonable to assume that user behaviors in other countries are different as well. Thus, like with other online phenomena [15, 16, 27], the context in which web search behavior is studied needs to be accounted for, and generalizations from single-country samples to the global populations should be avoided.

The fact that web search accounts for a rather high share of desktop browsing (around 13%), highlights the importance of search engines for the public. Similarly, the fact that top-5 results attract around 90% of all clicks and more than 97% of search visits do not go beyond page 1 of search outputs, underscores the influence search rankings have on user information consumption. The similarity of this finding to the observations regarding the result ranking made by previous studies over a decade ago [6, 7, 12, 19], underscores the persistence of the observed phenomenon. It can undoubtedly contribute to the "rich-gets-richer" bias and search concentration, but also stresses the importance of reliability and accuracy of the top results. Given that search engines’ retrieval and ranking algorithms are usually obscure and the outputs are heavily affected by personalization [8, 14, 23] and randomization [28], algorithmic auditing studies, with a particular focus on the ranking of top search results, are necessary to understand how specific factors affect the search outputs and the quality of information consumed by the users. Additionally, more research in the area of human–computer interaction assessing how design choices can help mitigating users’ bias towards top outputs—e.g., by employing grid design instead of ranked lists [14]—is necessary.

As web search algorithms tend to optimize the results based on user behavior [1], demographic and socio-economic status-based differences in the ways users search the web can have important implications for the selection of information that users access. For instance, if a search engine is used disproportionately more by users with certain characteristics, the search results will be tailored to the preferences of users with such characteristics. In extreme cases, this can lead to systematic biases in search results—e.g., the perpetuation of "male gaze" in results concerning the representation of women [19]. For this reason, we suggest more comparative analysis of search results provided by different search engines in countries where Google does not hold absolute dominance (e.g., Russia or South Korea) as well as the ways users interact with different engines are necessary to infer whether co-existence of multiple engines can contribute to the emergence of search engine-based filter bubbles and divergence in the information diets and beliefs of the audiences of different engines.

Our observations also have important implications for search engine design. The disproportionate amount of attention received by the top search outputs highlights the importance of giving consistent priority to reliable sources, in particular in the case of searches, where outputs might have substantive impact on individual or collective well-being (e.g., in the case of COVID-19-related information, but also, for instance, elections). It also stresses challenges for search diversification task by showing the limited space of outputs which can be meaningfully diversified. Finally, the discrepancies across countries and demographic groups (e.g., with regard to gender) indicate the potential for taking into consideration the local differences in search practices when designing regional interfaces for search engine (e.g., for ".ru" or ".de" versions of individual engines).

Finally, we would like to note that our study demonstrates the possibilities that a combination of web tracking and survey data offers for research in web search behavior. While this work is largely exploratory and only examined general search patterns, we suggest future work could utilize the opportunities offered by web tracking to also investigate the search queries utilized by different users when searching for information on similar topics—as previous findings indicate differences in search query formulations are connected, for instance, to users’ political attitudes [29]. As our findings highlight the contextual differences in general search behavior, we also suggest more cross-country investigations into web search behavior are warranted.

## Data Availability

Owing to the highly sensitive nature of the web tracking data, the data cannot be released publicly. Upon request, the aggregate data used to conduct the statistical analysis will be provided.
